# Integrated proteomics and metabolomics analysis reveals hubs protein and network alterations in myasthenia gravis

**DOI:** 10.18632/aging.204156

**Published:** 2022-07-08

**Authors:** Tong Tong, Jing Zhang, Li Jia, Ping Liang, Na Wang

**Affiliations:** 1 Department of Anesthesiology, The Fourth Affiliated Hospital of Hebei Medical University, Shijiazhuang 050011, China; 2 Department of Pharmacy, The Fourth Affiliated Hospital of Hebei Medical University, Shijiazhuang 050011, China; 3 Department of Gynaecology, The Fourth Affiliated Hospital of Hebei Medical University, Shijiazhuang 050011, China

**Keywords:** WGCNA, proteomics, metabolomics, gene co-expression networks, myasthenia gravis

## Abstract

Background: Thymoma-associated myasthenia gravis (TAMG) is a well-described subtype of Myasthenia gravis (MG). Nevertheless, the detailed proteins and bioprocess differentiating TAMG from TAMG (−) thymoma have remained unclear.

Methods: The proteomics and metabolomics were carried out on serum samples from thymoma group (*n* = 60, TNMG), TAMG (+) thymoma group (*n* = 70, TAMG (+)), and TAMG (−) thymomas group (*n* = 62, TAMG (−)), and controls (*n* = 159). groups. Proteomics and metabolomics analyses, including weighted gene co-expression network analysis (WGCNA), was conducted to detect the hub proteins and metabolomics processes that could differentiate TAMG (+) from TAMG (−) thymomas. MetaboAnalyst was used to examine the integration of proteomic and metabolomic analysis to differentiate TAMG (+) from TAMG (−) thymomas.

Results: The of module–trait correlation of WGCNA analysis identified KRT1, GSN, COL6A1, KRT10, FOLR2, KRT9, KRT2, TPI1, ARF3, LYZ, ADIPOQ, SEMA4B, IGKV1-27, MASP2, IGF2R was associated with TAMG (+) thymomas. In addition, organismal systems-immune system and metabolism-biosynthesis of other secondary metabolites were closely related to the mechanism of TAMG (+) pathogenesis.

Conclusion: Our integrated proteomics and metabolomics analysis supply a systems-level view of proteome changes in TAMG (+), TAMG (−) thymomas and exposes disease-associated protein network alterations involved in.

## INTRODUCTION

Myasthenia gravis (MG) is a chronic autoimmune disease resulting from autoantibodies (Abs) that target vital components of the neuromuscular junction (NMJ) on the postsynaptic membrane [1, 2]. MG is characterized by variable fatigable muscle disadvantage ranging from mild forms influencing bulbar muscles only to broad severe forms [3, 4]. It is a generalized syndrome that frequently exhibits primarily as focal weakness. Eye muscle weakness at the beginning of MG is obvious in a huge majority of sufferers leading to diplopia and ptosis [5, 6]. Corticosteroids, azathioprine, mycophenolate mofetil, and cyclosporine are widespread therapeutic strategies in treating myasthenia [7]. Whereas being operational in most MG patients, these immunotherapies are accompanied by damaging long-term effects, frequently unbearable for sufferers.

Thymoma is a neoplasm of the thymus, which is tightly associated with autoimmune disorders, particularly MG [8]. Additionally, numerous reports have confirmed that MG is characterized by autoantibodies against synaptic apparatus in the neuromuscular junction (NMJ) and classified into some subtypes [9]. About 85% of patients exhibits antibodies against the acetylcholine receptor (AChR). Thymus abnormalities take place in two subtypes of AChR+ MG patients, thymoma-associated (TAMG) and AChR+ early-onset form (EOMG). The subgroup associated with thymoma and nearly accordant with anti-acetylcholine receptor (AChR) antibody is termed as thymoma associated MG (TAMG). The incidence rate of TAMG in MG population is about 10–20%. As reported previously, approximately 35–50% of thymoma patients will eventually develop MG, whereas 10–15% of MG patients suffer from thymoma [2, 10]. Furthermore, in the early stages of this disorder, the anomalous functions and morphology of the thymus are commonly detected, which are characterized by thymic hyperplasia [11]. As an uncommon autoimmune disorder of the neurological system, the pathogenesis of MG remains largely unknown. Additionally, the specific proteins and metabolic processes that differentiate TAMG (+) from TAMG (−) thymomas remain largely elusive.

In the current study, the proteomics and metabolomics were carried out with the serum samples from thymoma patients, thymoma-associated MG (TAMG (+)) patients, thymoma without myasthenia gravis (TAMG (−)) patients, and controls. Weighted gene co-expression network analysis (WGCNA) was performed to explore the hub proteins and co-expressed modules which could differentiate TAMG (+) from TAMG (−) thymomas. In addition, MetaboAnalyst was used to examine the integration of proteomic and metabolomic analysis in MG with the potential to differentiate TAMG (+) from TAMG (−) thymomas.

## METHODS

### Clinical information

From May 2016 to May 2019, 132 adult (male 85, female 47, age 10–78, median 45 ± 7.8, duration 43.67 ± 50.59) MG patients were enrolled from the Fourth Affiliated Hospital of Hebei Medical University and performed with median follow-up of 24 months. Age of onset: 3 cases ≤18 years old, 13 cases aged 19~29, 35 cases aged 30 ~ 39, 26 cases aged 40 ~ 49, 45 cases aged 50 ~ 59, 9 cases aged 60 ~ 69 years and 1 case aged ≥70 years. MG participants included 53 patients who had myasthenia gravis with anti–acetylcholine receptor antibodies, 40 patients who had myasthenia gravis with MuSK antibodies, and 39 patients who had myasthenia gravis with Ryanodine receptor (RyR) antibodies. According to the modified Osserman classification for MG, patients were divided into: I (ocular muscle type) 9 cases, type IIA (mild systemic type) 27 cases, type IIB (moderate systemic type) 49 cases, type III (acute severe type) 27 cases, type IV (late onset severe type) 20 cases. 62 patients who had myasthenia gravis but no thymoma, 60 thymoma patients but no myasthenia gravis symptom and 159 controls healthy volunteers. The healthy volunteers were enrolled containing 101 males and 58 females during the same period with gender- and aged-matched to the MG patients. This study was approved by ethical committees of the Fourth Affiliated Hospital of Hebei Medical University. According to criteria in the incipient study, the MG sufferers were detected based on their medical history. The MG patients with the thymoma were verified by the pathological examination or imagery system. All in all, the participants were divided into four subgroups as follows: thymoma group (*n* = 60, TNMG), TAMG (+) thymoma group (*n* = 70, TAMG (+)), and TAMG (−) thymomas group (*n* = 62, TAMG (−)), and controls (*n* = 159).

### Proteomics

The proteomics analysis was performed by Applied Protein Technology Co., Ltd (Shanghai, China). The lysed samples were labeled with a TMT Kit (TMT 6plex, Thermo Scientific) and fractionated by RP-HPLC Scientific Q Exactive Focus Orbitrap LC-MS / MS System. Three hundred proteins were identified in at least 286 of the 298 serum samples among the four groups, including 45 of 48 serum samples of TNMG group, 49 of the 53 serum samples of the TAMG (+) group, 46 of the 48 serum samples of the TAMG (+) group, and 146 of 149 serum samples of the control group. These data were standardized using the intensity normalization methods.

### WGCNA

The WGCNA package for an R software package (http://www.r-project.org/) was applied to carry out a co-expression network of proteins, investigate the co-expressed modules, the correlation between the network and module–traits, and examine hub genes. WGCNA is a network approach focusing on gene sets other than single genes, alleviating the multiple measurement problem in microarray analysis. Scale-free topology fitting index R2 and the soft threshold are vital in constructing the scale-free network and obtaining the best-fit topology model. Firstly, define the best-fitting soft threshold. The soft threshold power was defined as 12, and scale-free topology R2 = 0.85 was selected to calculate the topological overlap matrix (TOM). Secondly, protein co-expressed modules were constructed. The Pearson correlation coefficient matrix was calculated among the co-expressed proteins, TOM, and hierarchical clustering plots were constructed using the best-fitting soft threshold. Calculate the eigenvector value (ME) of each module. A hierarchical clustering plot was carried out on the modules to obtain the co-expressed protein modules. The connotation between modules and different clinical traits was assessed to define the modules associated with the subtypes of MG.

### Construction of protein-protein interaction network (PPI)

String database (https://string-db.org) was used to predict the interaction between proteins. The intersection proteins were submitted into the string database. The threshold value of interaction was set at 0.4. The plug-in in Cytoscape v3.7.2 was used to screen the hub proteins selected in each module and depicted the PPI network.

### Metabolomics analysis

Targeted metabolomics was carried out according to their standardized analysis platform. The methods for sample preparation and GC-MS metabolomics analysis were conducted as described in an earlier report. Data analysis was performed with MarkerLynx (Waters Corp). We performed WGCNA and PPI networks to observe samples distributions and integrate metabolomics and proteomics data analysis.

### Functional enrichment analysis

Functional enrichment analysis was conducted on hug proteins and co-expressed modules by using Metascape. Go function annotation and KEGG pathway enrichment analysis were applied to conduct a comprehensive functional analysis of DEPs in co-expressed modules related to the disease. *P* < 0.05 was considered statistically significant.

### Statistical analysis

Unpaired Student’s *t*-test was employed to perform the two-group comparison. Unpaired Student’s *t*-test was used to test significance between genotypes where noted. One-way ANOVA followed by Tukey’s multiple comparisons tests were employed to assess differences between groups. Significant interactions were followed by post-hoc tests. A *p*-value <0.05 as well as |fold change| >2 was used. Data are represented as mean ± SEM. Data were processed using SPSS 19.0 (IBM) and GraphPad Prism software (version 1.0. San Diego, California).

### Availability of data and materials

The datasets used and analyzed during the current study are available from the corresponding author on reasonable request.

## RESULTS

### Hierarchical clustering and co-expressed modules identified by WGCNA

**Figure 1:**
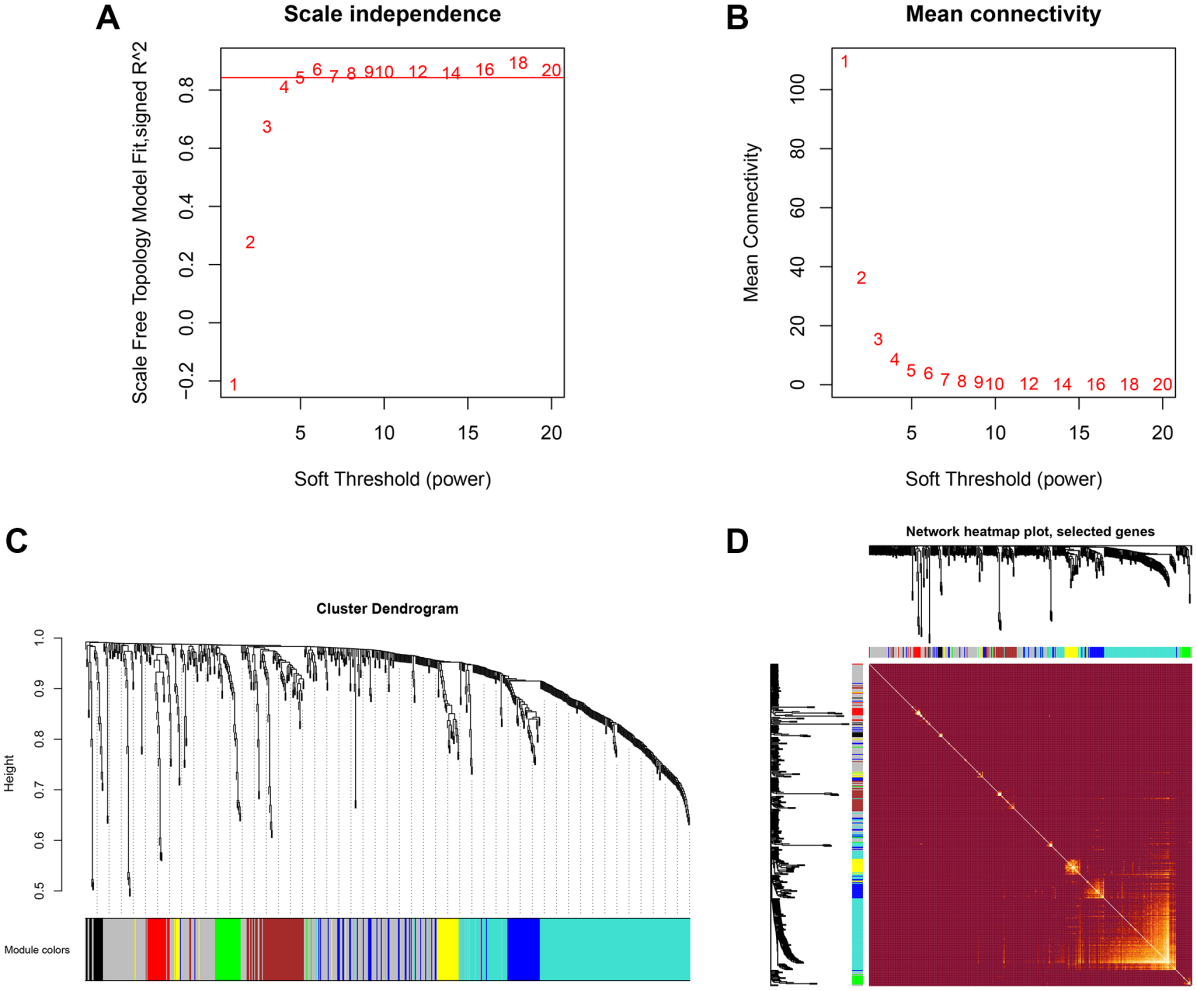
**Hierarchical clustering and co-expressed modules identified by WGCNA.** (**A**, **B**) the scale-free network, was created with the scale-free R2 = 0.8 and soft threshold = 10 to obtain the best-fit topology model. (**C**) 10 modules were identified based on the hierarchical clustering dendrogram of proteins. (**D**) The topological overlap map (TOM) for distinctive modules.

To explore the network of proteins involved in MG, co-expression modules of proteins and the correlation between modules and etiopathogenesis of MG were constructed by WGCNA. Three hundred proteins were identified at least 216 of 219 serum samples of TAMG (+), TAMG (−), TNMG, and control groups. WGCNA was employed to explore DEP modules and built the scale-free network based on scale-free topology R2 = 0.85, where the soft threshold power was defined as 10 to get the best-fitting topology model (Figure 1A and 1B). Based on the hierarchical clustering plot of differentially expressed intersection proteins,10 highly co-expressed modules were spotted, i.e., turquoise, green, magenta, grey, black, blue, brown, pink, red, and yellow modules (Figure 1C). The topological overlap map (TOM) of distinctive modules was demonstrated in Figure 1D.

### Modules strongly connected with TAMG (+) thymomas, TAMG (−) thymomas, and TNMG

**Figure 2:**
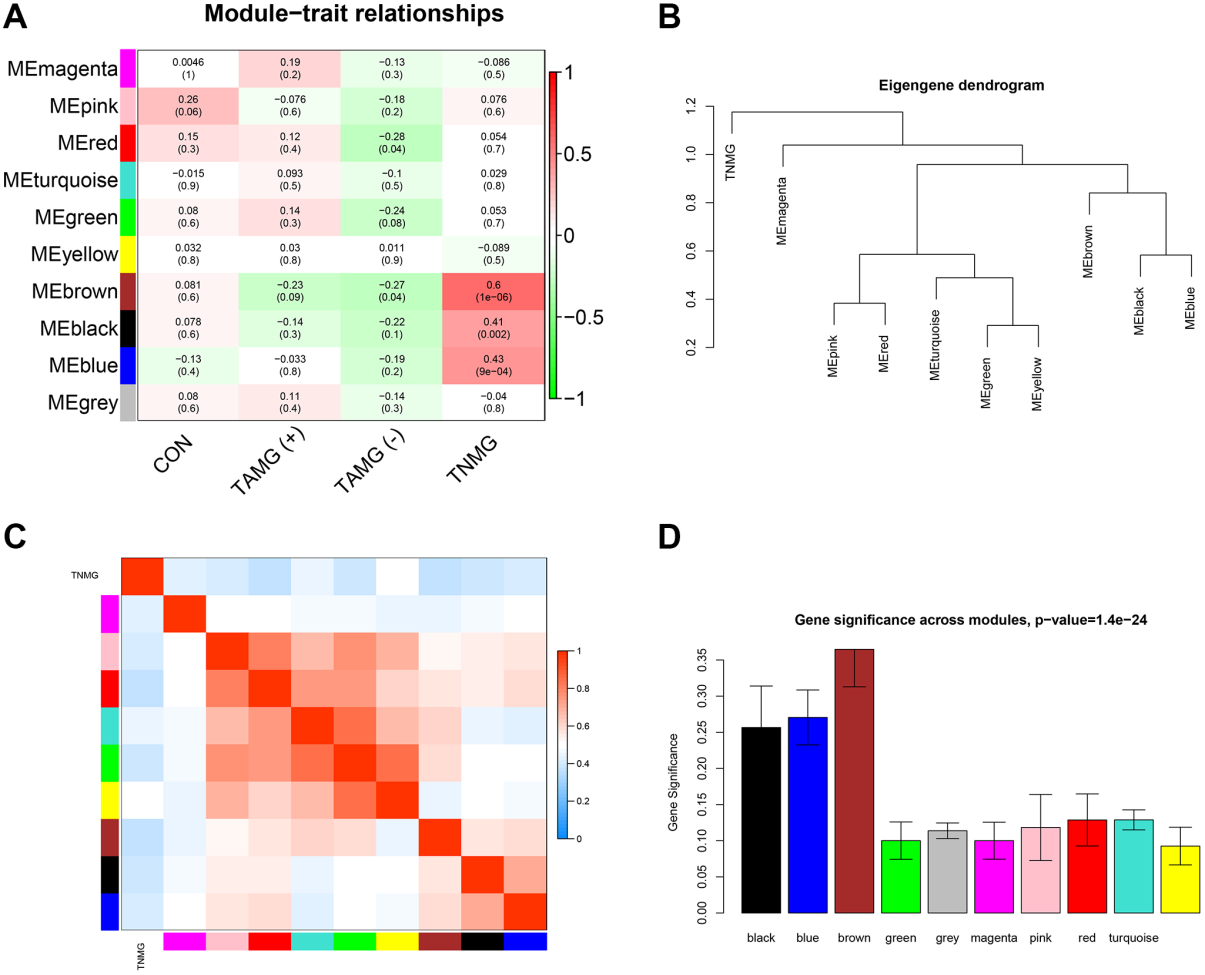
**Modules strongly connected with TNMG, TAMG (+), and TAMG (−).** (**A**) the module-trait correlation plot according to the clustering dendrogram of module eigengenes. (**B**) the module–module connection diagram. (**C**) analysis of the scale-free topology model. (**D**) protein co-expression modules with their module size.

The connection between modules and the disease phenotypes was assessed. As shown in the module–trait relationship diagram, the MEbrown module was highly associated with TNMG (Pearson correlation coefficient = 0.6). Furthermore, the MEbrown module (Pearson correlation coefficient = −0.23) was highly associated with TAMG (+) thymomas status, and the MEred module (Pearson correlation coefficient = −0.28) was highly associated with TAMG (−) (Figure 2A). Distinctive modules were differentiated according to the clustering dendrogram of module eigengenes (Figure 2B). The module–module connection diagram was depicted in Figure 2C. A histogram of the co-expressed modules’ significance demonstrated that the Brown module’s significance was the tallest amongst the modules (Figure 2D).

### Modules and hub proteins most relevant to myasthenia gravis

**Figure 3:**
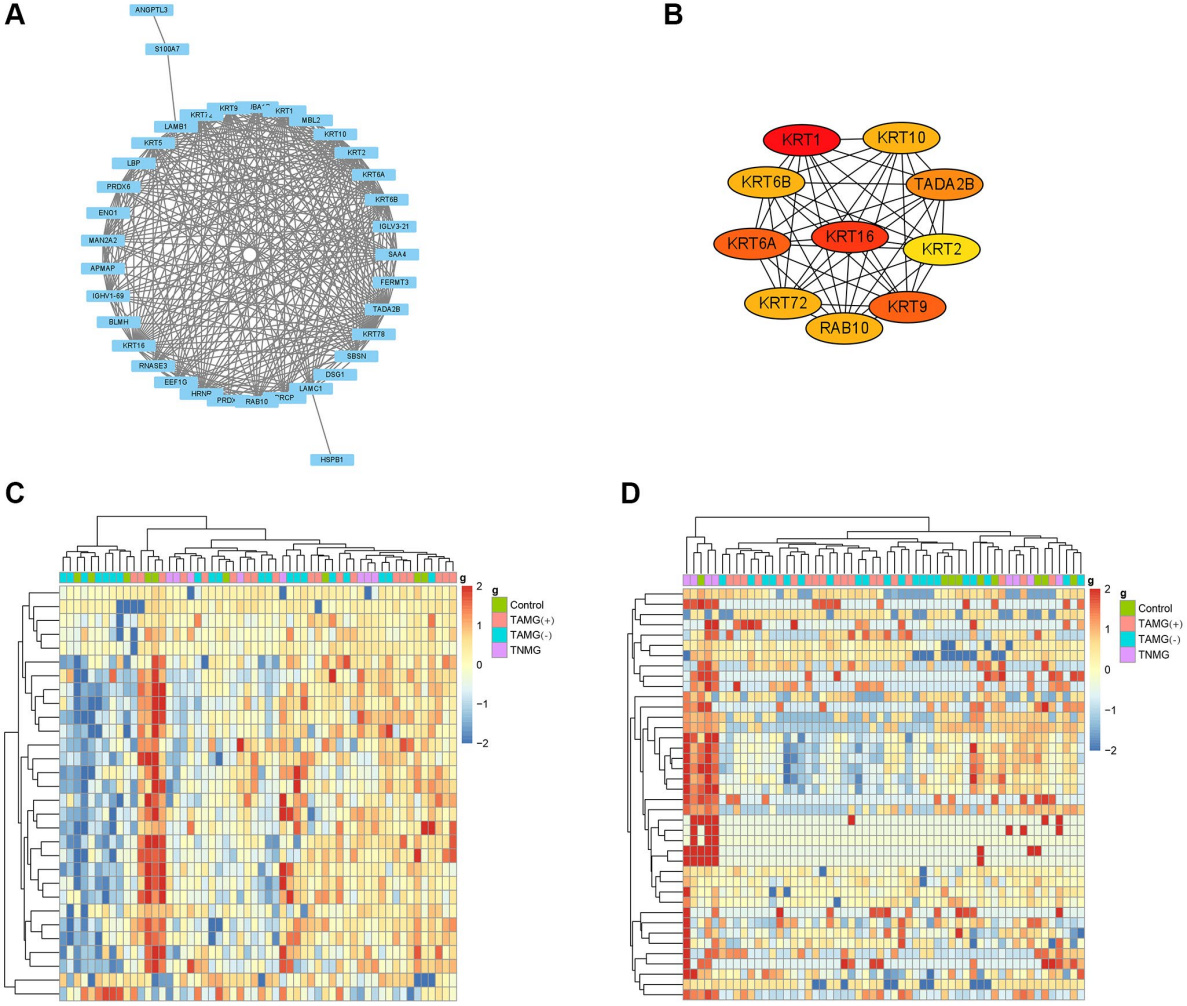
**Modules and hub proteins most relevant to myasthenia gravis.** (**A**) hub proteins in the TAMG (+)-related brown module were identified by the protein-protein interaction (PPI) network. (**B**) the top 10 strongly associated hub proteins for the identified TAMG (−)-related module. (**C**, **D**), the hierarchical clustering diagrams were depicted to exhibit the up- and down-regulated proteins relevant to TAMG (+) and TAMG (−).

Earlier work indicates that hub proteins in WGCNA modules have been considered essential biomarkers and guide treatment indicators. To illustrate the modules, we studied the biologic continuity among the proteins in the TAMG (+)-related brown module by plotting the hub proteins in the protein-protein interaction (PPI) network (Figure 3A). The results identified KRT1, GSN, COL6A1, KRT10, FOLR2, KRT9, KRT2, TPI1, ARF3, LYZ, ADIPOQ, SEMA4B, IGKV1-27, MASP2, IGF2R were associated with TAMG (+). The top 10 strongly associated hub proteins for the identified TAMG (−)-related module is exhibited in the network diagram (Figure 3B). Moreover, the hierarchical clustering diagrams were depicted to exhibit the up-and down-regulated proteins relevant to TAMG (+) (Figure 3C) and TAMG (−) (Figure 3D).

### Distinctions and connections in metabolomic profiles between TAMG (+) and TAMG (−)

**Figure 4:**
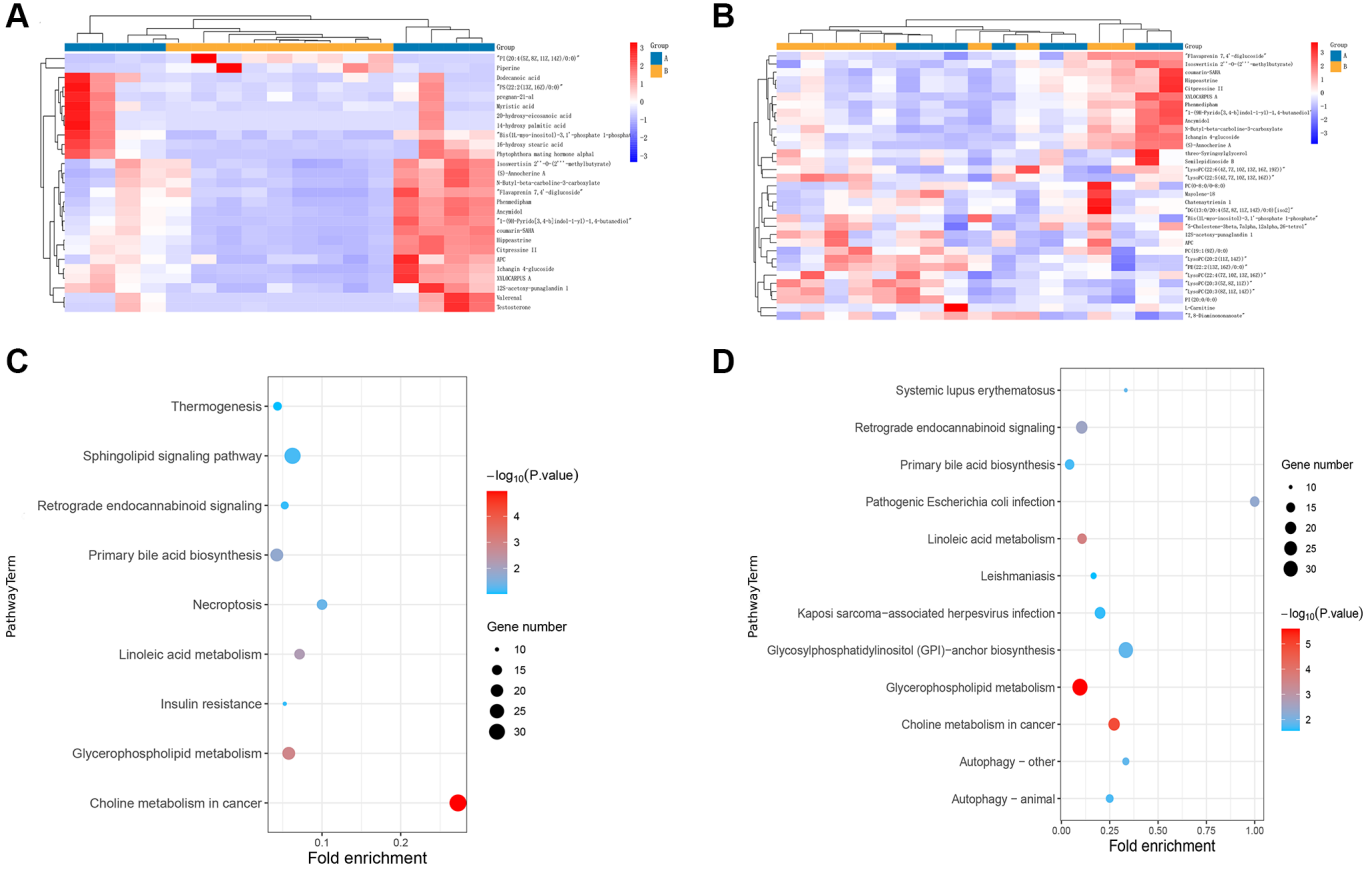
**Distinctions and connections in metabolomic profiles between TAMG (+) and TAMG (−).** (**A**, **B**) the hierarchical clustering diagrams for the metabolites. (**C**, **D**) pathway analysis for metabolomic profiles.

We next carried out non-targeted serum metabolomics to investigate the metabolic impact in myasthenia gravis. Compared with the control group, 350 and 377 metabolites identifiers were statistically different in TAMG (+) (Figure 4A) and TAMG (−) (Figure 4B), respectively. The enrichment analysis exposed that the metabolomic profiles were implicated in Sphingolipid signaling pathway, Glycerophospholipid metabolism, Choline metabolism in cancer, Linoleic acid metabolism, Fatty acid degradation, Histidine metabolism, Primary bile acid biosynthesis, Nicotinate and nicotinamide metabolism, and Glycine in both TAMG (+) and TAMG (−) (Figure 4C and 4D).

### Integration of proteomic and metabolomic analysis

**Figure 5:**
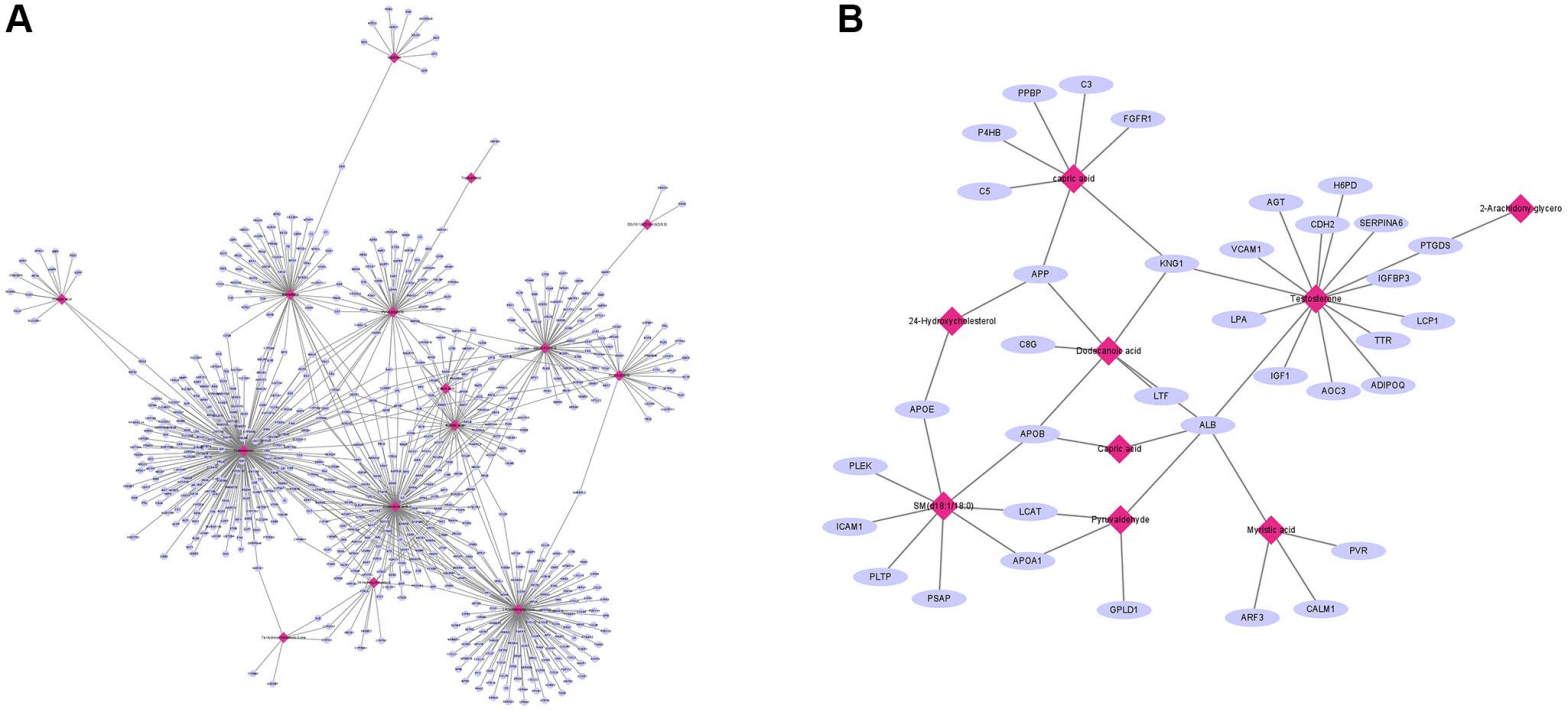
**Integration of proteomic and metabolomic analysis.** (**A**) MetaboAnalyst was used to explore the association between proteomic and metabolites. (**B**) hub metabolites for both the TAMG (+) and TAMG (−) networks.

We further explored the association between proteomic and metabolites through MetaboAnalyst. For TAMG (+) thymomas, 194 differently expressed proteomics and 350 metabolites were identified and composed of a network with 152 nodes (proteomics and metabolites) and 180 edges (interactions) (Figure 5A). Meanwhile, another network of 41 nodes and 20 edges was created between the differently expressed proteomics and metabolites in TAMG (+) thymomas (Figure 5B). The networks were composed through Cytoscape software. We found that Dodecanoic acid, 2-Arachidonyl glycerol, Pyruvaldehyde, 24-Hydroxycholesterol are hub metabolites for both the TAMG (+) thymomas and TAMG (−) thymomas networks.

### GO analysis of the significant MEmagenta and MEred modules in the TAMG (+) thymomas and TAMG (−) thymomas groups

**Figure 6:**
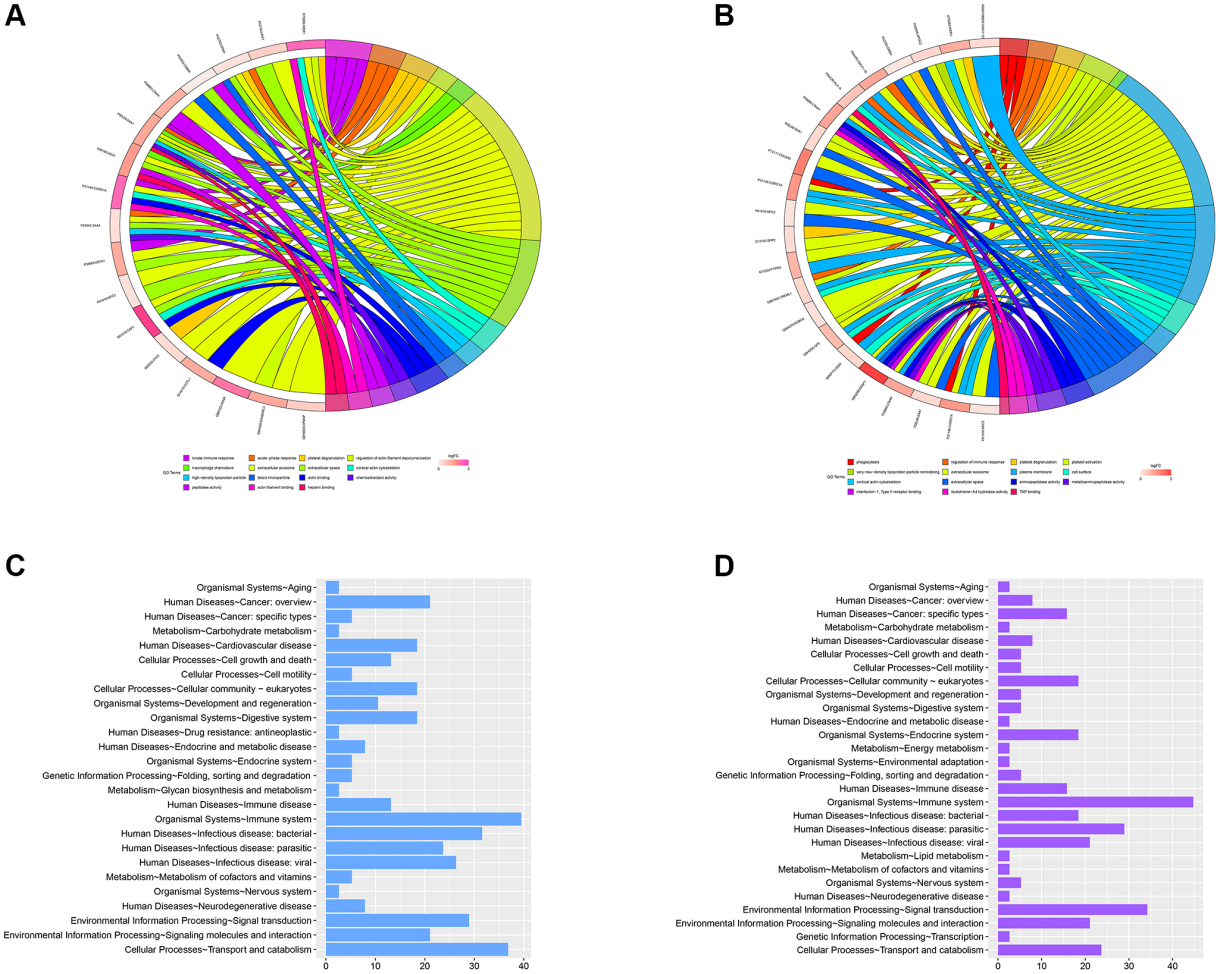
**GO analysis of the significant MEmagenta and MEred modules in the TAMG (+) and TAMG (−) groups.** (**A**, **B**) GO annotation for module’s DEPs. (**C**, **D**) KEGG pathway analysis of MEmagenta module.

GO annotation exhibited that the MEmagenta module’s DEPs were mainly differentially gathered in extracellular exosome, blood microparticle, adaptive immune response, antigen binding, and receptor-mediated endocytosis processes in TAMG (+) thymomas (Figure 6A). Similarly, the DEPs, associated with the MEred module were mainly gathered in extracellular exosome, receptor-mediated endocytosis, antigen binding, platelet degranulation, and complement activation in TAMG (−) thymomas (Figure 6B).

In addition, KEGG pathway analysis of the MEmagenta module demonstrated that proteins were mainly differentially gathered in cellular processes-cell growth and death, metabolism-amino acid metabolism, metabolism-biosynthesis of other secondary metabolites, human diseases-Endocrine and metabolic disease, and organismal systems-immune system (Figure 6C). Meanwhile, KEGG pathway analysis of the MEmagenta module was demonstrated in Figure 6D.

To the end, organismal systems-immune system and metabolism-Carbohydrate metabolism were closely related in the mechanism of TAMG (+) thymomas and TAMG (−) thymomas pathogenesis, and both might be underlying approaches for the hindrance and healing of TAMG (+) thymomas and TAMG (−) thymomas.

## DISCUSSION

Myasthenia gravis (MG) is a neuromuscular disorder with antibodies directed against the skeletal muscle nicotinic acetylcholine receptor (AChR), the muscle specific kinase (MuSK) [12]. Considerable progress has been made in exploring the onset and therapeutic improvement. However, a third of patients still suffer MG aggravations and react badly to standard treatment, which requires hospitalization, and morbidity remains high [13]. Current epidemiology indicates that, similar to other autoimmune disorders, the prevalence of MG is growing noticeably [14]. MG is closely related to the thymus, and thymic abnormalities, such as thymoma, accompany patients with MG [15, 16]. Thymoma sustains intra-tumorous thymopoiesis to an alterable level and relates to the TAMG (+) thymoma occurrence. In TAMG, the target is often the acetylcholine receptor (AChR).

In recent years, proteomics and metabolomics analysis have been progressively applied to offer innovative views to treat MG. Gomez et al. indicate that transformations in cytoplasmic proteins are implicated in the pathogenesis of MG [17]. Serum proteomic and metabolomic analyses were performed with myasthenia gravis patients to explore the therapeutic effect of Qiangji Jianli Fang on MG [18]. Derrick et al. show that metabolomic profiling distinguishes patients with MG disease from healthy persons. The metabolomic analysis might offer a critical biomarker for refining MG characterization [19]. Meanwhile, another study confirmed that metabolomic identifications could be recognized as prednisone responsive biomarkers to develop diagnostic correctness and forecast therapeutic consequences in MG [20]. Tan et al. indicate that distinctive subtypes of MG might produce discriminatory fecal metabolism [21]. Although proteomic and metabolomic identifications are widely applied in the study of TAMG, a detailed understanding of the integration analysis of proteomic and metabolomic in TAMG (+) is limited. Differences in integrating proteomic and metabolomic profiles between TAMG (+), TAMG (−) and thymoma, and the interactions with proteomic and metabolomic data remain unknown.

This study applied the serum protein data from TAMG (+), TAMG (−), thymoma and control candidates to depict co-expression modules’ protein–metabolite interaction. WGCNA analysis identified co-expression modules of proteins and the correlation between modules and etiopathogenesis. Three hundred proteins were identified in at least 216 of 219 serum samples of TAMG (+), TAMG (−), TNMG, and control groups. Based on the hierarchical clustering plot of differentially expressed intersection proteins, ten highly co-expressed modules were spotted. The topological overlap map (TOM) identified the distinctive modules. The of module–trait correlation of WGCNA analysis identified KRT1, GSN, COL6A1, KRT10, FOLR2, KRT9, KRT2, TPI1, ARF3, LYZ, ADIPOQ, SEMA4B, IGKV1-27, MASP2, IGF2R was associated with TAMG (+) thymomas. In addition, organismal systems-immune system and metabolism-biosynthesis of other secondary metabolites were closely related to the mechanism of TAMG (+) pathogenesis. Based on our non-targeted serum metabolomics, 350 metabolites identifiers were statistically different in TAMG (+) and TAMG (−) groups. GO enrichment analysis of these modules showed that the metabolomic profiles were implicated in Sphingolipid signaling pathway, Glycerophospholipid metabolism, Choline metabolism in cancer, Linoleic acid metabolism, Fatty acid degradation, Primary bile acid biosynthesis, Nicotinate and nicotinamide metabolism, and Glycine in both TAMG (+) and TAMG (−). Extensive studies have shown that linoleic acid supplementation might have therapeutic potential concerning insulin sensitivity and lipid metabolism [22]. Furthermore, linoleic acid is an efficient mediator in constraining the progression of hyperinsulinemia [23]. By integrating distinctive types of MG data, we could explore more novel insights than those studied individually. Capric acid, Dodecanoic acid, Pyruvaldehyde, and 24-Hydroxycholesterol in the protein–metabolite interaction network indicate that they might have notable and essential roles in the pathogenesis of GB. In addition, cellular processes-cell growth and death, metabolism-amino acid metabolism, metabolism-biosynthesis of other secondary metabolites, metabolism-Carbohydrate metabolism, human diseases-Endocrine, and organismal systems-immune system signaling pathways were strongly activated in the disease mechanisms. Most importantly, the organismal systems-immune system and metabolism-Carbohydrate metabolism were closely related in the mechanism of TAMG (+) thymomas and TAMG (−) thymomas pathogenesis, and both might be underlying approaches for the hindrance and healing of TAMG (+) thymomas and TAMG (−) thymomas. Indeed, the immune system is a multifaceted system containing local and specific tissue spots related to circulating immune cells. The innate immune system is the front line of host defense. Innate immune cells are activated by danger signals, including pathogen and danger-related molecular patterns, and metabolite-related danger signals. The disorder of the immune system is vital for the origination and development of chronic inflammatory diseases, including TAMG. Notably, metabolic flexibility is crucial to preserve vitality homeostasis and relies on the conformation of metabolic pathways. Metabolic flexibility or metabolic inflexibility is associated with many pathological conditions, including TAMG.

In conclusion, our integrated proteomics and metabolomics analysis supply a systems-level view of proteome changes in TAMG (+), TAMG (−) thymomas and exposes disease-associated protein network alterations involved. The identified co-expressed modules network and their hub proteins create novel comprehensions of TAMG (+) and TAMG (−) thymomas. We believe that the proteomics and metabolomics analysis is helpful to further understand the pathogenesis of MG.
